# Lateralization difference in functional activity during Stroop tasks: a functional near-infrared spectroscopy and EEG simultaneous study

**DOI:** 10.3389/fpsyt.2023.1221381

**Published:** 2023-08-23

**Authors:** Zemeng Chen, Xiang Ji, Ting Li, Chenyang Gao, Guorui Li, Shuyu Liu, Yingyuan Zhang

**Affiliations:** ^1^Institute of Biomedical Engineering, Chinese Academy of Medical Sciences and Peking Union Medical College, Tianjin, China; ^2^Applied Physiology and Kinesiology, College of Health and Human Performance, University of Florida, Gainesville, FL, United States; ^3^Academy of Opto-Electronics, China Electronics Technology Group Corporation, Tianjin, China

**Keywords:** lateralization difference, functional near-infrared spectroscopy, electroencephalogram, brain connectivity analysis, event-related potential, Stroop task

## Abstract

**Introduction:**

Conflict monitoring and processing is an important part of the human cognitive system, it plays a key role in many studies of cognitive disorders.

**Methods:**

Based on a Chinese word-color match Stroop task, which included incongruent and neutral stimuli, the Electroencephalogram (EEG) and functional Near-infrared Spectroscopy (fNIRS) signals were recorded simultaneously. The Pearson correlation coefficient matrix was calculated to analyze brain connectivity based on EEG signals. Granger Causality (GC) method was employed to analyze the effective connectivity of bilateral frontal lobes. Wavelet Transform Coherence (WTC) was used to analyze the functional connectivity of the bilateral hemisphere and ipsilateral hemisphere.

**Results:**

Results indicated that brain connectivity analysis on EEG signals did not show any significant lateralization, while fNIRS analysis results showed the frontal lobes especially the left frontal lobe play the leading role in dealing with conflict tasks. The human brain shows leftward lateralization while processing the more complicated incongruent stimuli. This is demonstrated by the higher functional connectivity in the left frontal lobe and the information flow from the left frontal lobe to the right frontal lobe.

**Discussion:**

Our findings in brain connectivity during cognitive conflict processing demonstrated that the dual modality method combining EEG and fNIRS is a valuable tool to excavate more information through cognitive and physiological studies.

## 1. Introduction

Conflict monitoring and processing has always been a key characteristic of the human cognitive system. It is closely related to the study of neurological disorders, stroke, and congenital cognitive dysfunction in children (1, 2). The Stroop task is one of the most widely used methods for studying how the human brain detects and resolves conflicts (3, 4). In 1935, John Ridley Stroop first discovered that when the meaning of a printed word was different from the color of the word, there would be a cognitive delay, which is the Stroop effect (5). The Stroop effect makes behavioral responses to incongruent stimuli (when the word’s meaning and the word’s ink color are not consistent, e.g., the word “blue” shown in red ink) less accurate and slower than responses to neutral stimuli (6) (when the word’s meaning and the word’s ink color are consistent).

In recent years, there have been several functional neuroimaging methods applied to detect brain activity during the Stroop task, such as Electroencephalogram (EEG) and functional Near-infrared Spectroscopy (fNIRS) (7, 8). As to EEG, it records neutral activities with a high temporal resolution, which is within the millisecond range, but has a lack of spatial resolution resulting from volume conduction, thus leading to barriers in source localization (9, 10). The past few decades have seen a rapid increase in the use of fNIRS for monitoring metabolic change in the cerebral cortex, which has excellent spatial but low temporal resolution resulting from the inherent hemodynamic delay (11, 12). Moreover, fNIRS is more robust than EEG when confronted with motion-based muscle activity and electrical noise artifacts (13). The dual-modal imaging technology that combines the high spatial resolution of fNIRS with the high temporal resolution of EEG has gained attention (14–17). EEG-fNIRS correlation analysis helped to further reveal the complex relationship between electrophysiological and hemodynamic changes in neuroscience (18).

As for EEG, Event-related potentials (ERP) analysis was employed to trace the time course of different executive processes and sub-processes involved in experimental tasks involving a cognitive conflict with precision (19). In previous studies, the ERP frontal-central N200 component reflects conflict monitoring procedures (20, 21). The Pearson correlation coefficient (PCC) is an indicator of brain functional connectivity, which is widely accepted during the measurement of the statistical relationships between random variables and the relationships between signals (22). As to fNIRS, a previous study has used Dynamic Causal Modeling (DCM) to analyze effective connectivity, and the used general linear model (GLM) to identify brain regions that are significantly activated during the Stroop task (23). Another study employed Granger Causality (GC) to evaluate the effective connectivity between hemispheres, and Wavelet Transform Coherence (WTC) to assess intrahemispheric functional connectivity (15). This study proved that GC and WTC analysis can be reliable methods for fNIRS data processing. One previous study initially recorded the EEG signals and fNIRS data simultaneously, they used ANOVA analysis on N450 ERP and hemodynamic activation data. And the result indicated that N450 reflects conflict detection and resolution, the left frontal lobe may be involved in conflict detection, and the bilateral frontal lobe is engaged in conflict resolution (15).

In this study, we aimed to find out which brain region plays the leading role, in local information processing, and how information flow transfers inside the human brain in processing Stroop tasks. A Chinese color-word match Stroop task was employed since it primarily activates the bilateral frontal lobes (24–26). The behavioral data, EEG signals from the whole brain, and fNIRS signals from the frontal lobe and the parietal lobe were recorded simultaneously. The GC method was employed to analyze the effective connectivity of bilateral frontal lobes. The WTC method was used to analyze the functional connectivity of the bilateral hemisphere and ipsilateral hemisphere. According to the results, we found out that the left frontal lobe plays the leading role during processing the more complex incongruent tasks. Meanwhile, the human brain showed leftward lateralization, which means the ability of local information processing in the left frontal region increased, and the information flow from the left frontal lobe to the right frontal lobe also increased. This study made us gain a deeper understanding of brain activity during cognition processing.

## 2. Materials and methods

### 2.1. Participants

A total of 21 healthy volunteers (9 females/12 males, aged 23.0 ± 2.3 years ranging from 20 to 30 years) were recruited from the Institution of Biomedical Engineering, Peking Union Medical College. All participants’ color vision is normal. and they have no history of mental illness or other related diseases that may interfere with the conclusion of the study. All participants are Chinese native speakers, and the whole experiment paradigm was performed in a Chinese environment. Experimental procedures involving human subjects described in this paper were approved by the Clinical Research Ethics Committee of the First Affiliated Hospital, College of Medicine, Zhejiang University (IIT20210036C-R1). All participants signed informed consent before the experiments and were compensated for their effort by being given a certain amount of test fee.

### 2.2. Procedures

**Figure 1:**
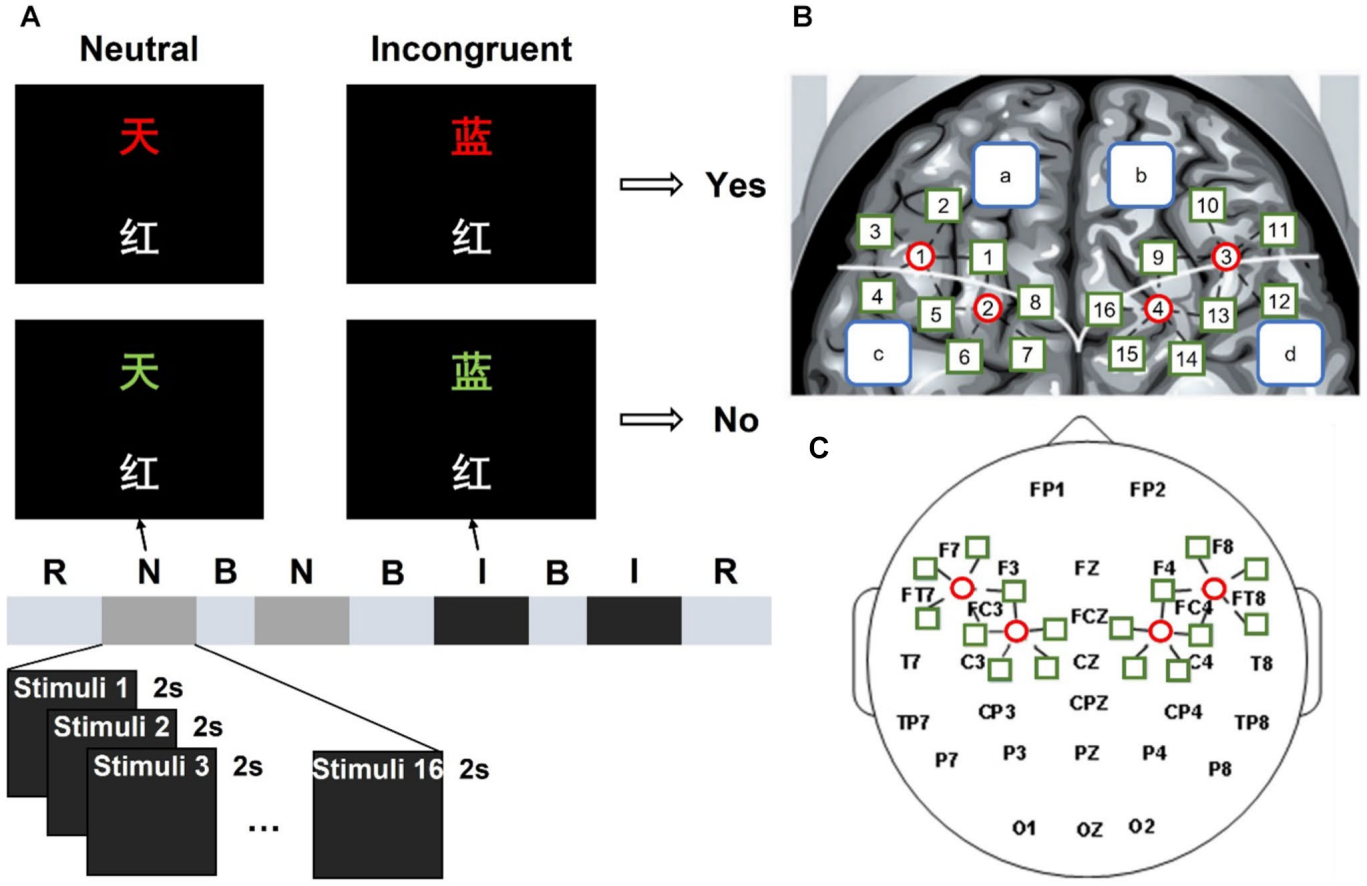
Experimental process and position arrangement of the fNIRS and EEG probe channel of the system. **(A)** “R” is short for rest, “N” is short for the neutral stimulus block, “B” is short for break, and “I” is short for the incongruent stimulus block. **(B)** Left and right fNIRS channel number and brain region distribution. **(C)** fNIRS probe site with the 10–20 system.

The classical verbal Stroop color-word task with a block design was employed in this study, which was adapted from a previous study (19). Each stimulus contained two Chinese characters and participants were asked to judge whether the color of the upper one matched the meaning of the lower one, as shown in Figure 1A. If the two Chinese characters in the stimulus were corresponding, participants pressed the left mouse button and held it until a trial ended. On the contrary, participants pressed the right button. There were two different kinds of stimulus conditions: neutral and incongruent. In neutral stimuli, the upper Chinese character was a noncolor word which consists of “贯,” “奖,” “放,” “社,” meaning “pass through,” “prize,” “lay,” and “society,” presented in red, yellow, blue, or green; the lower Chinese character was a color word which consists of “红,” “黄,” “蓝,” “绿,” meaning “red,” “yellow,” “blue,” and “green,” presented in white. In incongruent stimuli, the upper Chinese character was a color word presented in a different color. For each stimulus, the numbers of “corresponding” trails and “not corresponding” trails were equal, and those two trails were randomly mixed within each block.

Four blocks were included in this study, they were displayed in order: neutral stimulus block, neutral stimulus block, incongruent stimulus block, and incongruent stimulus block. Each block consisted of 16 trials. During each trial, a stimulus was presented for 2 s, and there are 5 s intervals between two successive trials. There were 30 s rest periods before the first block and after the fourth block. During the break and interval, a white cross was shown in the center of the screen with a black background. A beep sound appeared 0.5 s before each block to remind participants. There was a practice session before the formal experiment to let participants are familiar with the task. The experimental environment was kept dark and quiet to minimize disturbance to the participants.

### 2.3. Data recording

As for behavioral data, the judgment results and the reaction time of all participants for each stimulus are collected and recorded by the stimulus program.

Figure 1C shows the 10–20 system of the international federation, which was used for EEG recording as the scalp site, with the left mastoid using a Neuroscan 64-channel device (Synamps) to record, while the right mastoid was as a reference. The Electrooculograms (EOGs) were recorded using four additional bipolar electrodes. Two electrodes were placed in the superior and inferior areas of the left orbit to record vertical EOG, and two electrodes were placed lateral to the left and right orbits to record horizontal EOG. A 0.05–100 Hz band-pass filter was designed for the EEG and EOG data, then a 50 Hz notch filter is used. The sampling rate of the continuous records is 100 Hz. Electrode impedances were kept under 5 kΩ.

This study used a continuous-wave, modulated light source fNIRS system developed by our laboratory to record the fNIRS data (27). It consists of continuous wave laser diodes (L785P100, Thorlabs, USA and ADL85501TL, Roithner Lasertechnik, Austria), direct digital synthesis chip (ML2035, Micro Linear, USA), micro control unit (AT89C2051, Atmel, United States), custom-made dual fiber collimators (Space Star Aerospace Technology Applications Co., Ltd., China), and avalanche photodiode (C5460-01, Hamamatsu Photonics, Japan). Based on the modified Beer–Lambert Law, two wavelengths (785 nm and 850 nm) were used to determine the concentration changes of HbO_2_ (Δ[HbO_2_]) and Hb (Δ[Hb]). The fNIRS probe was placed in the EEG electrode cap and held four sources (represented by the red circle in Figure 1B) and 18 detectors (represented by the green square in Figure 1B), providing 20 detector channels covering the frontal and parietal lobes. For example, S1-D1 is short for light source 1 and detector 1 is a channel. In Figure 1B, the white curve divided the frontal and parietal lobes into two parts, the blue squares “a” and “b” represented the left and right frontal lobe respectively, and the blue squares “c: and “d” represented the left and right parietal lobe, respectively. There were 10 detector channels in both the left and right regions with a 3 cm interval. The sampling rate of our fNIRS system is 100 Hz.

During the experiment, the brightness of the lower right corner of the screen was changed with the change of the type of stimuli, which made a photoelectric marking module generate different signals for the fNIRS system and EEG system through a capture card. This made optical and electrical signals can be detected simultaneously, and it was also useful in data processing for data alignment.

### 2.4. Data analysis

#### 2.4.1. Behavioral data analysis

MATLAB™ R2022b (MathWorks, Natick, MA, United States) was used to process the raw behavioral data, and statistical tests were analyzed using IBM Statistics SPSS 24. Accuracy, reaction time, and accuracy divided by reaction time (accuracy/reaction time) were calculated for both incongruent stimulus and neutral stimulus. The paired *t*-test was conducted for paired accuracy, reaction time, and accuracy/reaction time.

#### 2.4.2. EEG data analysis

This study used the EEG data processing toolbox developed by André Mouraux (Institute of Neuroscience, Université catholique de Louvain, Belgium) et al.^1^ The EEG data were Butterworth band-pass filtered with a frequency range from 0.05 to 30 Hz. If the electrode with excessive impedance was recorded according to the experiment, the bad electrode was removed and interpolated. Independent component analysis (ICA) matrix was computed, and after that, the movement artifacts and eye-blink artifacts were identified and removed by applying ICA spatial filter. The EEG data were then segmented into 1,000-ms epochs, including a 200-ms pre-stimulus baseline. The segmented EEG data were re-referenced to channel CZ data. After the baseline correction, epochs were averaged separately for incongruent stimulus and neutral stimulus.

##### 2.4.2.1. ERP analysis

Based on our results and previous studies, analyses focused on N200 and P300. Both N200 and P300 ERP components in EEG signals, can be detected during cognitive tasks to executive cognitive control functions (28), and response to word presentation (29). Each peak N200 amplitude and peak P300 amplitude was extracted for both the incongruent and neutral stimuli. The N200 amplitudes for P7 and P8 channels and P300 amplitudes for CP3 and CP4 channels for each stimulus were extracted. Then paired *t*-tests were used for paired data.

##### 2.4.2.2. Brain connectivity analysis of EEG signals

The PCC values of EEG channels within left and right hemispheres (EEG channels in left hemisphere: P3, P7, CP3, TP7, C3, T7, FC3, FT7, F3, F7, FP1. EEG channels in right hemisphere: P8, P4, TP8, CP4, T8, C4, FT8, FC4, F8, F4, FP2) for both stimuli were calculated to analyze the brain connectivity to provide more information about the lateralization differences using EEG signals. A MATLAB function was applied to calculate the PCC values between every 11 channels with half hemisphere. Then average PCC values were calculated and the mean intra-hemispheric connectivity matrix was plotted, separately based on EEG signals between subjects in the incongruent and neutral stimulus. Each matrix element was the average PCC value for an EEG channel pair represented by its row coordinate and column coordinate.

#### 2.4.3. fNIRS data analysis

In this study, HbO_2_ was employed to estimate the changes in cerebral blood oxygenation because HbO_2_ has a better signal-to-noise ratio (SNR) than Hb (30).

According to the marker generated by the photoelectric marking module, raw fNIRS data of the incongruent stimulus and neutral stimulus were divided into two parts. A 3 Hz lower-pass filter was used first to the data to remove instrument noise, then downsampled to 10 Hz. After converting to a change in optical density, a ranged from 0.015 to 0.2 Hz band-pass filter was used to reduce gradual drifts and oscillations of the arterial pulse. Later on, the differential pathlength factor (DPF) method was applied to convert the optical intensity data into HbO_2_ and Hb signals, while the DPF values were 5.2 at 850 nm and 6.0 at 785 nm (30). The wavelet minimum description length detrending algorithm was used to suppress unknown global trends. At last, the HbO_2_ and Hb signals were stimuli averaged.

##### 2.4.3.1. Brain activation analysis

The mean value of HbO_2_ and Hb signals during the task period (0–40 s after the task began) were calculated for the task hemodynamic response for each channel. Then paired *t*-tests were conducted for paired HbO_2_ and Hb data for each channel.

##### 2.4.3.2. Effective connectivity analysis of bilateral frontal lobes

One of the brain connection analyses is the effective connection method. The causal relationship between the interactions of different brain regions is reflected by their analysis of effective connectivity. The analysis results describe how information flow transmit between different brain regions. The GC mathematical model was used to analyze effective connectivity between hemispheres (31, 32). Signal A can be referred to as the “Granger cause” of signal B if the past values of signal A can be utilized as *a priori* knowledge to forecast signal B. An autoregressive model was employed to calculate GC.

To explore the hemispheric lateralization properties of the brain processing different kinds of stimuli. GC values were calculated for HbO_2_ signals of corresponding channels between the frontal lobes of the left and right hemispheres (e.g., S1-D1 and S3-D9 are corresponding channels). The Difference of Influence (DOI) between brain regions was defined as the GC value for each channel pair with the left pointing to the right minus the GC value for the right pointing to the left. Therefore, a positive DOI value indicates that information flowed from the left-sided to the right-sided. To display the Granger causality properties between left and right brain regions, DOI values were obtained for four channel pairs in frontal regions. DOI values were calculated for incongruent and neutral stimuli.

This study used a Granger causal connectivity analysis MATLAB toolbox (Granger causal connectivity analysis, GCCA) developed by Seth (33). The Granger output results were affected by the lag period selection of the GC model. In this study, it was automatically selected according to the Bayesian information criteria (34), when, at their minimal values, these criteria indicate the best trade-offs between accuracy and parameter count. Therefore, the results are robust to variations in the model order. Then paired *t*-test was conducted to the mean DOI values of parameters with an automatically selected lag in the frontal lobes of the left and right hemispheres in the incongruent and neutral stimulus.

##### 2.4.3.3. Functional connectivity analysis of bilateral hemisphere

Functional connectivity undirected showed the statistical correlation of activity in different regions of the brain from the viewpoint of functional integration, using the WTC method in this study (31, 35). The WTC method indicated the time-frequency cross-correlation of two data series (36). It can also detect local phase information which might be difficult to find out by traditional time series analysis methods (37). In this study, a MATLAB wavelet transforms coherence toolbox developed by Grinsted et al. (38)^2^ was used to analyze the functional connectivity of bilateral and ipsilateral hemispheres.

The correlation analysis of the time series of HbO_2_ signals between corresponding channel pairs in the left and right hemispheres (e.g., S1-D1 and S3-D9 are corresponding channel pairs) was calculated using the WTC. The mean values of coherence value in the 0.071–0.5 Hz band (corresponding to period 14 and 2 s, respectively) were computed to indicate functional connectivity for each corresponding channel pair (37). The Fisher-z transform was used on the coherence values, then paired *t*-tests were performed on each channel pair in the incongruent and neutral stimulus.

##### 2.4.3.4. Functional connectivity analysis of ipsilateral hemisphere

The WTC analysis was used for correlation analysis of the time series of HbO_2_ signals between any two channels in the left and right hemispheres (L1-L10, R1-R10. L1 to L5 correspond to S1-D1 to S1-D5, L6 correspond to S2-D1, L7-L10 correspond to S2-D5 to S2-D8. R1 to R5 correspond to S3-D9 to S3-D13, R6 correspond to S4-D9, R7-R10 correspond to S4-D13 to S4-D16.). The mean values of coherence value in the 0.071–0.55 Hz band were computed to present the functional connectivity strength within the ipsilateral hemisphere, and this also came out with the left and right intra-hemispheric functional connectivity matrices. Then we plotted the mean intra-hemispheric functional connectivity matrix, separately based on HbO_2_ signals between subjects in the incongruent and neutral stimulus. Each matrix element was the coherence value for a fNIRS channel pair represented by its row coordinate and column coordinate. After the Fisher-z transform was used on the coherence values, paired *t*-tests were used for paired samples (39).

## 3. Results

The test results in the figure or table as followed abided the standards: *indicates *p* < 0.05, **indicates *p* < 0.01, ***indicates *p* < 0.001.

### 3.1. Behavioral results

**Figure 2:**
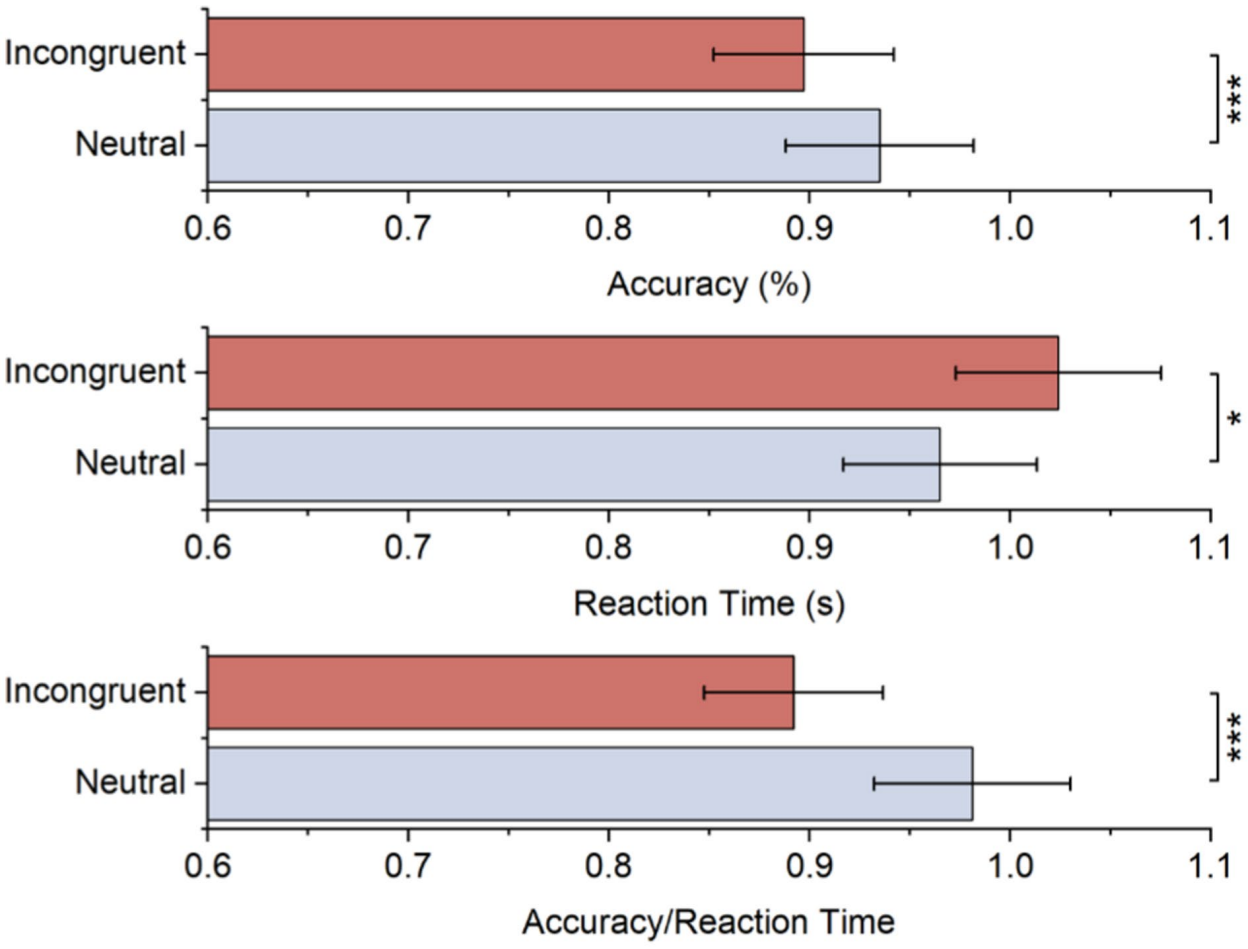
Accuracy, reaction time, and accuracy/reaction time for each stimulus.

Table 1 and Figure 2 displays the average accuracy, reaction time, and accuracy/reaction time for two stimulus conditions. As to accuracy, there was a significant difference between both stimulus conditions, and it was lower for the incongruent condition than the neutral condition. These behavioral results also suggested longer reaction times for incongruent conditions, while the difference was significant. Moreover, the accuracy/reaction time for the incongruent condition is significantly lower. All behavioral results showed significant Stroop effects.

**Table 1:** Statistical results of accuracy, reaction time, and accuracy/reaction time for each stimulus.

N	Value	Incongruent (M ± SD)	Neutral (M ± SD)	*t*
21	Accuracy (%)	0.897 ± 0.792	0.935 ± 0.051	2.737***
	Reaction time (s)	1.024 ± 0.120	0.965 ± 0.984	−4.282*
	Accuracy/Reaction time	0.892 ± 0.157	0.981 ± 0.133	4.227***

“*t*” is the *t-*value of paired *t*-test.

### 3.2. EEG results

#### 3.2.1. ERP results

**Figure 3:**
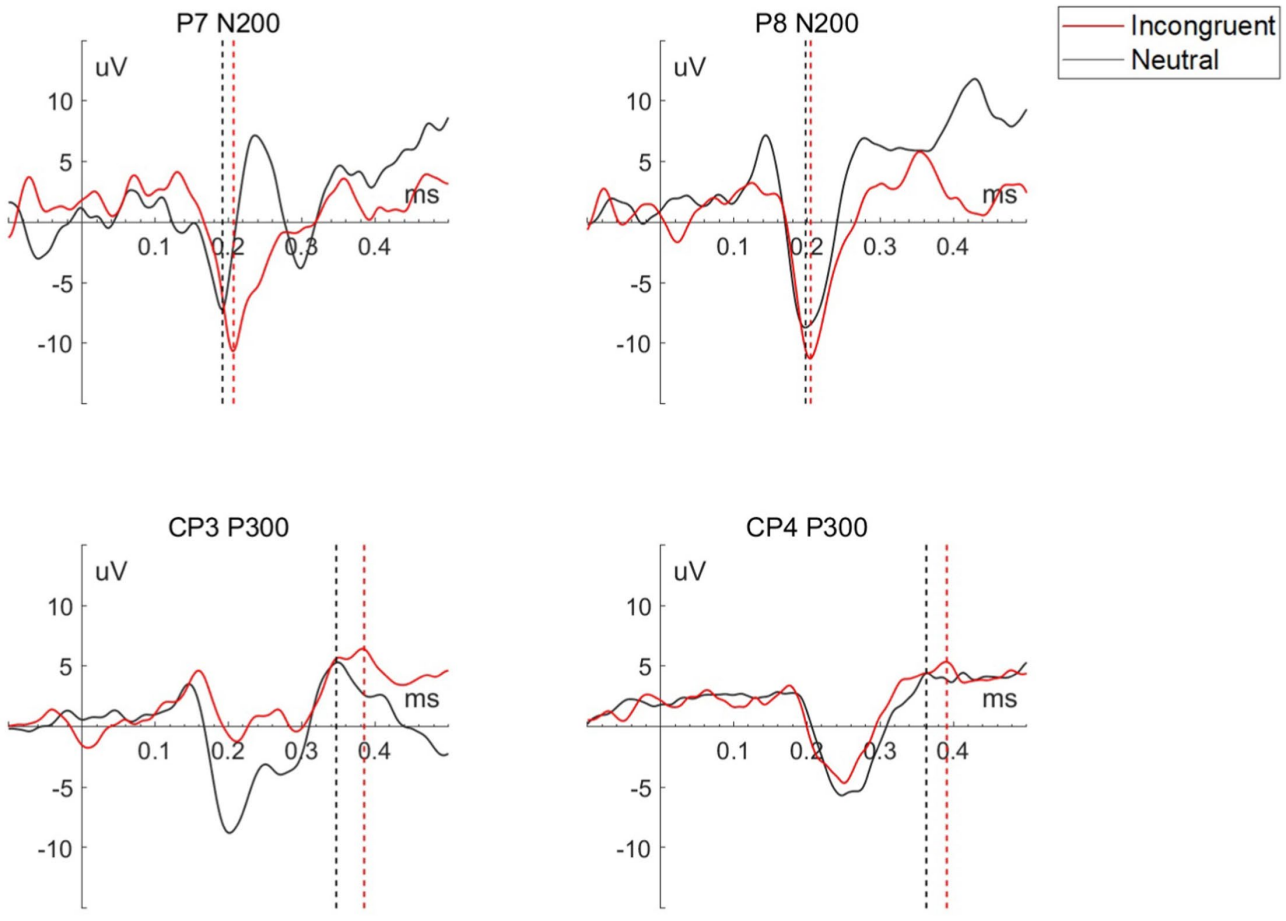
Waveform of P7 N200, P8 N200, CP3 P300, and CP4 P300 for each stimulus of a representative participant, the straight dashed line perpendicular to the x-axis marks the peak of each wave.

As shown in Table 2 and Figure 3, the two feature-based components, N200 in P7 and P8 channel, and P300 in CP3 and CP4 channel, presented significant differences for the incongruent and neutral stimulus. For incongruent stimulus, the occurrence time of both N200 and P300 in each channel was significantly longer than in neutral stimulus, while the peak of both N200 and P300 in each channel was significantly higher than in neutral stimulus.

**Table 2:** Statistical results of occurrence time and amplitude of P7 N200, P8 N200, CP3 P330, and CP4 N200 for each stimulus.

Channel	N	Task	Occurrence time (ms)	Amplitude (uV)	*t*
P7 N200	23	Incongruent	0.219	−14.960	−2.393*
		Neutral	0.198	−10.414	
P8 N200		Incongruent	0.220	−13.184	−2.513*
		Neutral	0.201	−9.446	
CP3 P300		Incongruent	0.381	6.992	−2.387*
		Neutral	0.365	5.808	
CP4 P300		Incongruent	0.376	6.518	−2.387*
		Neutral	0.366	5.613	

“*t*” is the *t*-value of paired *t*-test.

#### 3.2.2. EEG brain connectivity analysis results

**Figure 4:**
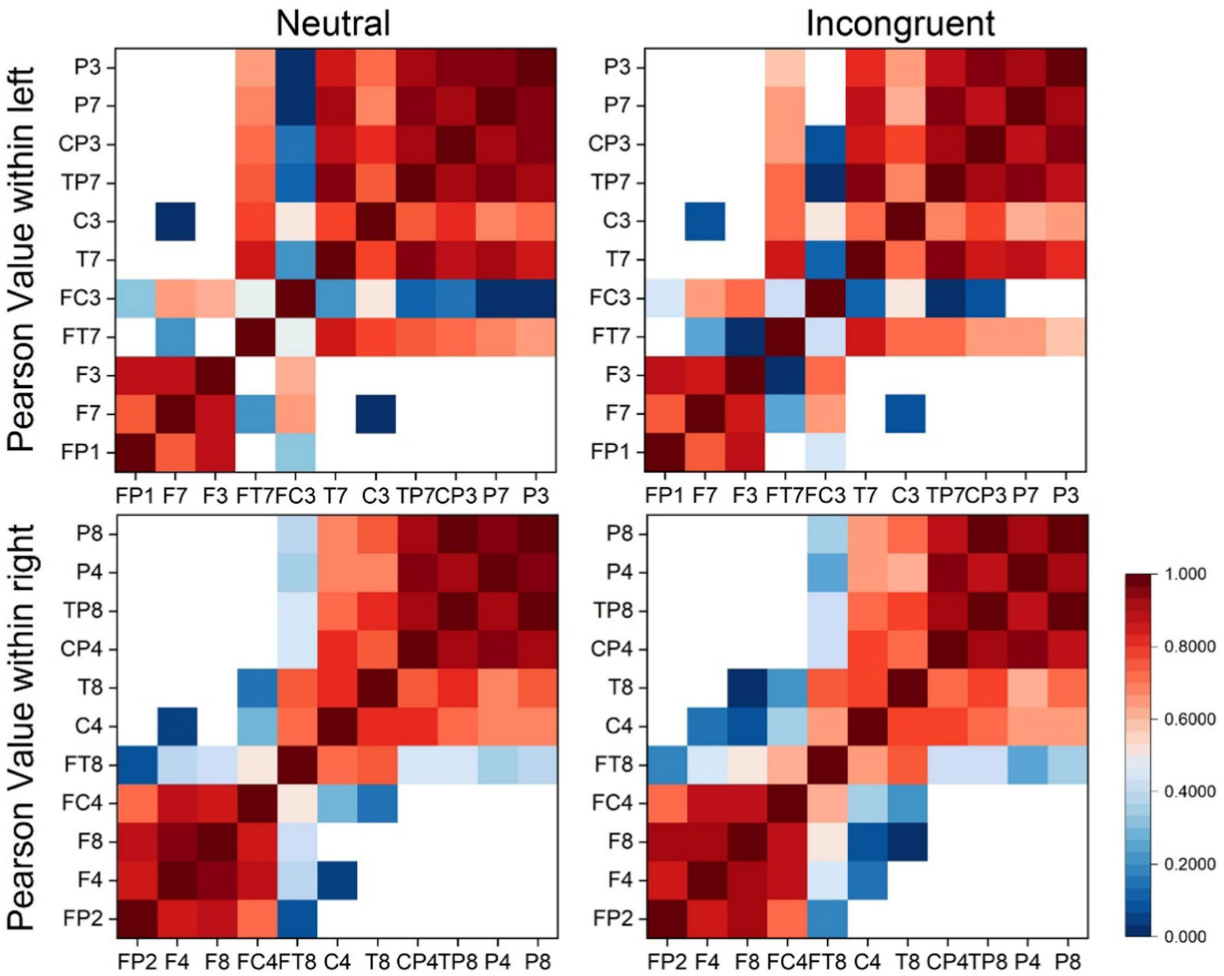
Brain connectivity matrix based on EEG signals.

Every matrix element of the EEG brain connectivity matrices represented the averaged PCC value between channel pairs (horizontal coordinates and vertical coordinates) as shown in Figure 4. According to the matrices, the PCC value of the left and right hemispheres had no big differences in both incongruent and neutral stimulus. What’s more, there were no significant differences between left and right hemispheres both in incongruent and neutral stimulus.

### 3.3. fNIRS results

#### 3.3.1. Brain activation analysis results

**Figure 5:**
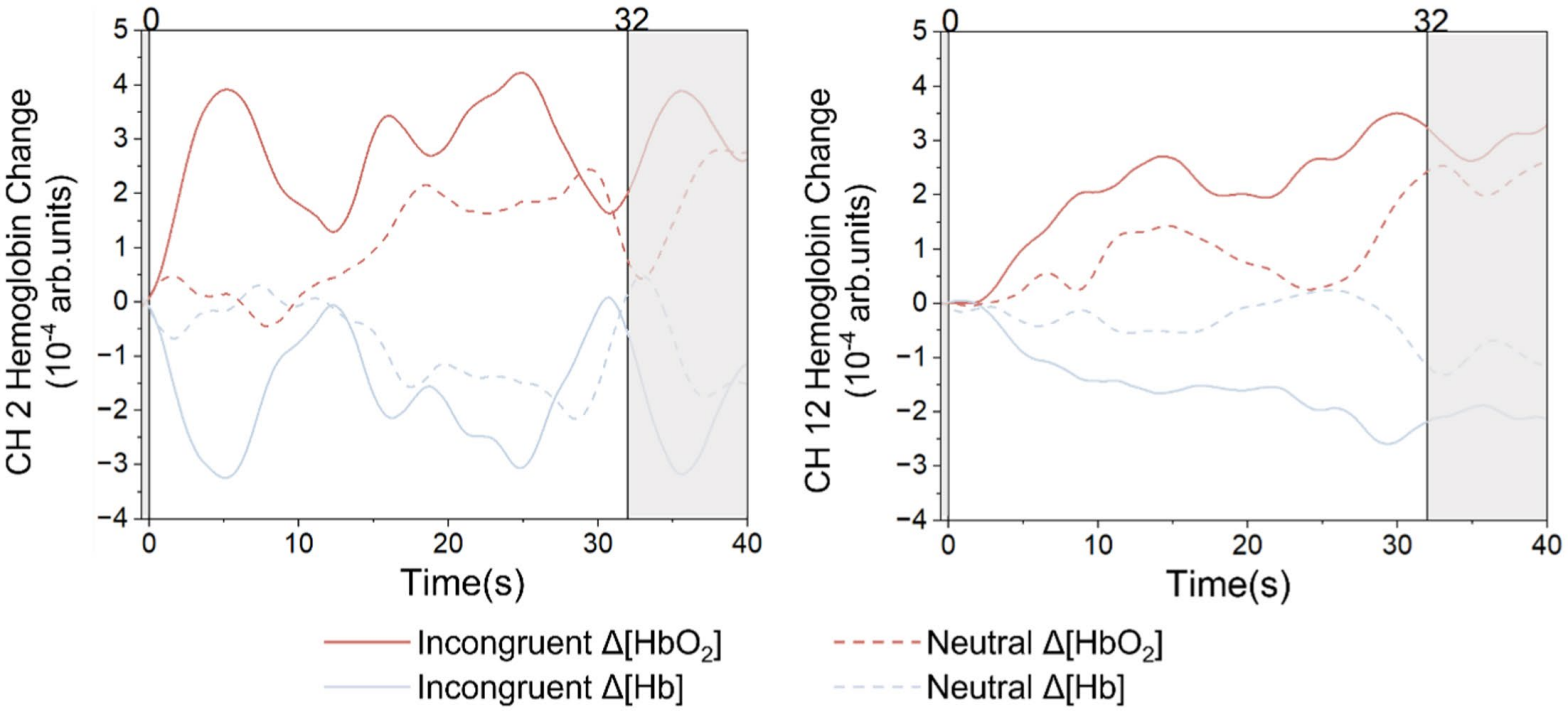
The grand average HbO_2_ and Hb signal for each stimulus.

Figure 5 shows the grand average HbO_2_ and Hb signals for the incongruent task and the neutral task at two typical frontal lobe channels. The two straight lines perpendicular to the x-axis mark the beginning and the end of the stimuli. In both S1-D2 and S3-D10, the activation responses for the incongruent task were significantly greater than the neutral task. The statistical results of HbO_2_ in S1-D2 (*t* = 2.499, *p* = 0.029), S3-D10 (*t* = 2.246, *p* = 0.024) showed significant differences. The statistical results of Hb on S1-D2 (*t* = 2.148, *p* = 0.033), S3-D10 (*t* = 3.118, *p* = 0.017) also showed significant differences.

#### 3.3.2. Results of effective connectivity analysis of bilateral frontal lobes

**Figure 6:**
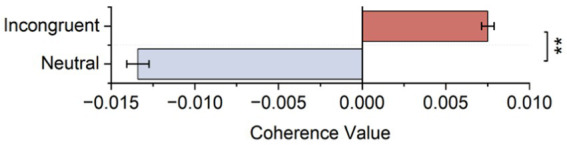
Mean DOI values in lag 4 s calculated based on HbO_2_ signals.

The automatically selected lag period for incongruent and neutral stimuli is 4 s. Meanwhile, the DOI value in the left and right frontal lobes of the incongruent stimulus was positive, and the DOI value in the left and right frontal lobes of the neutral stimulus was negative, as shown in Figure 6. Statistical tests showed significant differences in DOI values between incongruent and neutral stimulus at the lag period of 4 (*t* = 3.947, *p* = 0.001). The results showed that the information flow from the left to the right frontal lobes was major in accompanying conflict processing.

#### 3.3.3. Results of functional connectivity analysis of bilateral hemisphere

**Figure 7:**
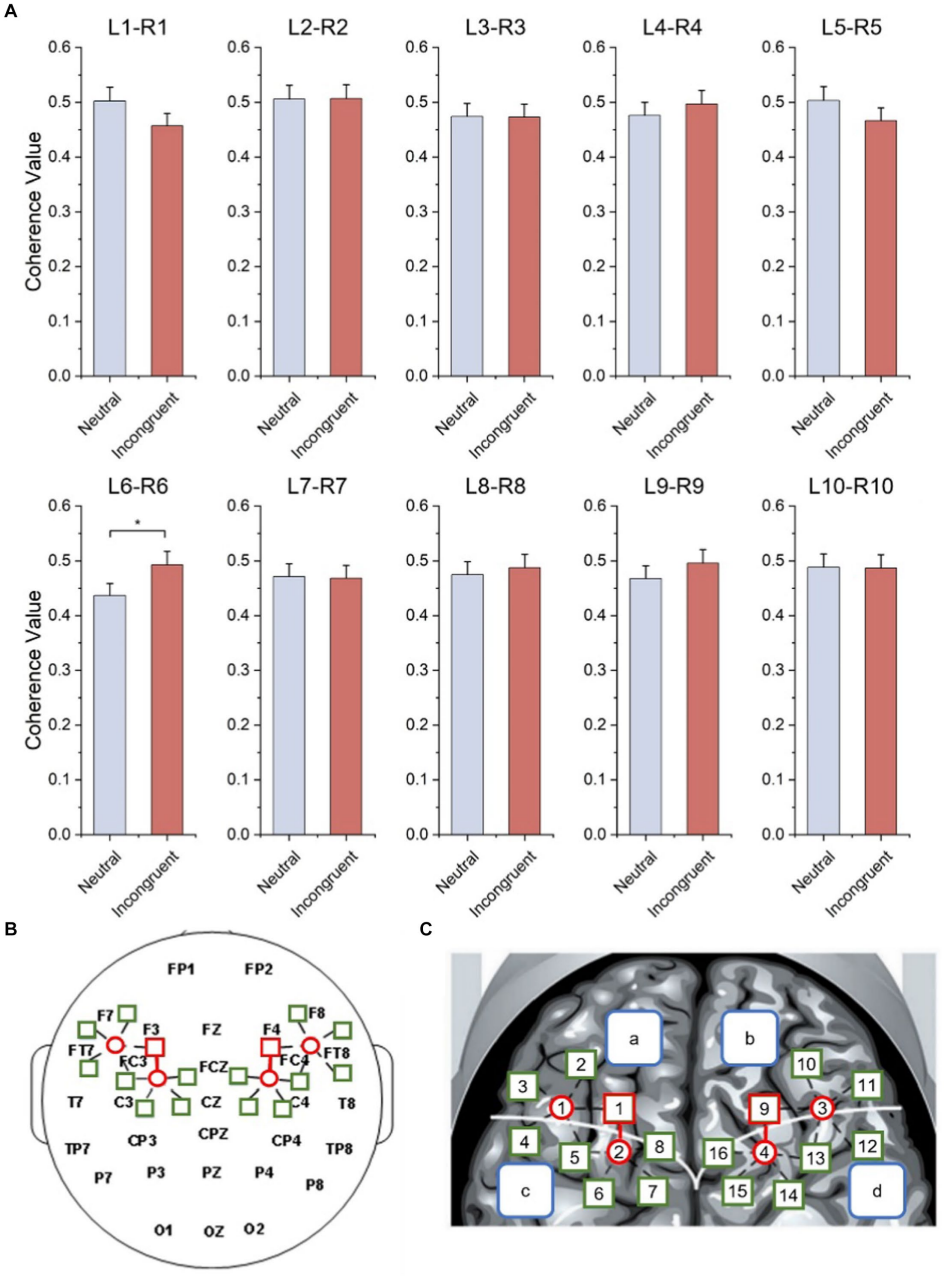
Bilateral hemisphere functional connectivity analysis results. **(A)** Average coherence values between left and right frontal paired channels based on HbO_2_ signals. **(B)** Channels with significant differences located in left and right fNIRS channel number and brain region distribution figure. **(C)** Channels with significant differences located in fNIRS probe site with the 10–20 system figure.

As shown in Figure 7A and Table 3, statistical tests show that in channel pairs L6 and R6, the strength of brain functional connectivity of incongruent stimulus was significantly stronger than the strength of brain functional connectivity of neutral stimulus (*t* = 2.617, *p* = 0.017). They were highlighted with red boxes in Figures 7B,C, and they were located in the frontal lobe.

**Table 3:** Statistical results of functional connectivity analysis of bilateral hemisphere.

Channel	Task	Coherence value	*T*	*p*
L1-R1	Incongruent	0.457	1.695	0.106
	Neutral	0.503		
L2-R2	Incongruent	0.507	−0.039	0.970
	Neutral	0.506		
L3-R3	Incongruent	0.473	0.039	0.969
	Neutral	0.474		
L4-R4	Incongruent	0.497	−0.648	0.524
	Neutral	0.476		
L5-R5	Incongruent	0.504	1.175	0.254
	Neutral	0.467		
L6-R6	Incongruent	0.493	2.617*	0.017
	Neutral	0.437		
L7-R7	Incongruent	0.469	0.118	0.907
	Neutral	0.471		
L8-R8	Incongruent	0.488	−0.447	0.660
	Neutral	0.475		
L9-R9	Incongruent	0.496	−0.902	0.378
	Neutral	0.468		
L10-R10	Incongruent	0.487	0.056	0.956
	Neutral	0.489		

“Coherence Value” is the average coherence values between left and right frontal paired channels based on HbO_2_ signals, “*t*” is the *t-*value of paired *t*-test, and “*p*” is the value of *p* of the paired *t*-test.

#### 3.3.4. Results of functional connectivity analysis of ipsilateral hemisphere

**Figure 8:**
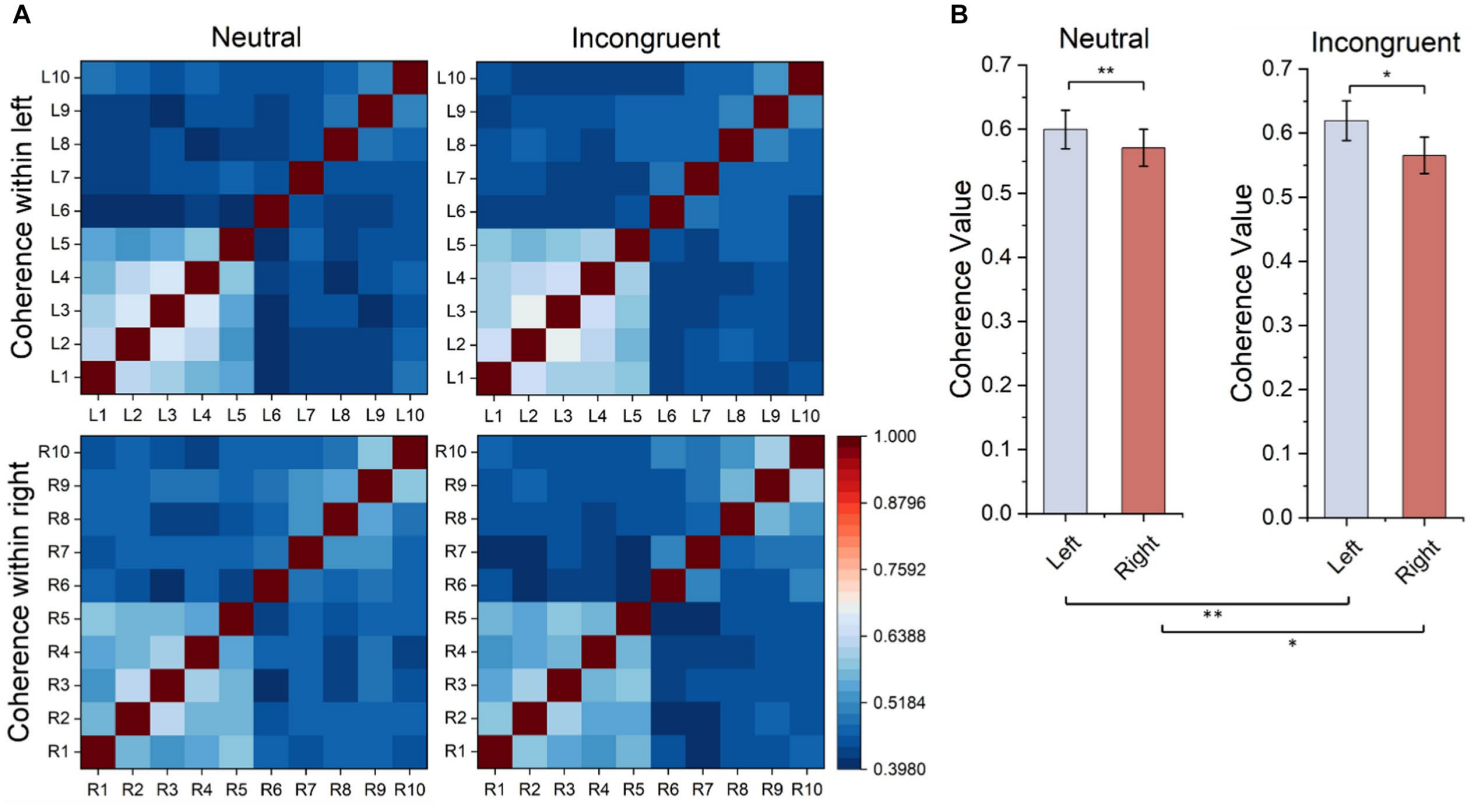
Ipsilateral hemisphere functional connectivity analysis results. **(A)** Ipsilateral hemisphere functional connectivity matrix based on HbO_2_ signals. **(B)** Average functional connectivity within left frontal lobes based on HbO_2_ signals.

Every matrix element of the functional connectivity matrices represented the correlation value between channel pairs (horizontal coordinates and vertical coordinates) as shown in Figure 8. According to the matrices, the frontal lobes had stronger functional connectivity than other areas of the brain, and the functional connectivity of the left frontal lobes is stronger than the functional connectivity of the right frontal lobes both in the incongruent and neutral stimulus. Therefore, statistical tests were employed for frontal lobes (L1–L5 and R1–R5) in both stimuli, and the functional connectivity within the left frontal lobes was significantly stronger than the functional connectivity within the right frontal lobes (neutral, *t* = 3.385, *p* = 0.001, incongruent, *t* = 2.326, *p* = 0.029). What’s more, the functional connectivity within the left frontal lobes of the incongruent stimulus was significantly stronger than that of the neutral stimulus, and the functional connectivity within the right frontal lobes of the incongruent stimulus was also significantly stronger than that of the neutral stimulus (left, *t* = 6.162, *p* = 0.001, right, *t* = 3.768, *p* = 0.020).

## 4. Discussion

In this study, we recorded behavioral data, EEG signals, and fNIRS signals simultaneously during the Chinese color-word matching Stroop task. The behavioral data performance, the EEG results, and the fNIRS activation results represented significant Stroop effects, indicating that our experimental design was correct, and making it reliable to find out about the internal information flow of the human brain when processing cognitive tasks.

Accuracy and accuracy/reaction time were significantly lower for the incongruent conditions than the neutral conditions, while the reaction time is the opposite, which showed significant Stroop effects. The two feature-based EEG components N200 and P300 also displayed significant Stroop effects. As for the N200, it can be detected when the human brain deals with cognitive tasks such as flanker tasks (40) and go/no-go tasks (41). The N200 is considered to be associated with cognitive control (28), making it explainable that, when the human brain processes the more complex incongruent stimulus, both the occurrence time and the peak of the N200 were larger than the neutral stimulus. The P300 is another event-related potential associated with cognitive processing. Our results showed that the occurrence time and the peak of the P300 for the incongruent conditions were significantly bigger than the neutral conditions, which were consistent with previous studies (42). The Chinese color-word matching Stroop task was employed to activate the bilateral frontal lobes (24, 43, 44). The fNIRS activation results showed that the amplitude of HbO_2_ signals in the frontal lobe for incongruent stimulus was significantly higher than for neutral stimulus, which indicated significant Stroop effects in both the left and right frontal lobes. These results are consistent with previous studies (24), representing that the recording of fNIRS data with high quality was correct.

The effective connectivity analysis of bilateral frontal lobes results in the 4 s lag DOI values in the left and right frontal lobes of the incongruent stimulus was positive and showed significant differences in DOI values between incongruent and neutral stimulus. As for the DOIs’ definition is the causality in the left-to-right direction minus that in the right-to-left direction (33), which corresponded to the net causality of the left to the right direction (45), indicated that for the incongruent stimulus versus the neutral stimulus, the information flow from the left frontal lobe to the right frontal lobe increased, demonstrating increased interhemispheric functional integration along with the attentional processing. According to one previous cognition research on healthy people, the flow of information from the left to the right prefrontal cortexes increased accompanying conflict processing (15)_._

The functional connectivity analysis of the bilateral hemisphere showed that the average coherence value between L6-R6 channel pair in congruent stimulus is significantly higher than in neutral stimulus, indicated that when the human brain processes more complex cognitive tasks like incongruent stimuli, the strength of brain functional connectivity between bilateral frontal lobes became stronger. Previous studies pointed out that interhemispheric functional integration was involved during attentional processing (46), and interhemispheric functional integration led to superior performance (47). According to the functional connectivity analysis of the ipsilateral hemisphere, the functional connectivity within the left frontal lobe in both incongruent and neutral stimuli is significantly stronger than the right frontal lobe, and the functional connectivity within the left frontal lobe of the incongruent stimulus is significantly higher than the neutral stimulus.

This study indicated that the combination of EEG and fNIRS is meaningful for the exploration of cognitive functions. One previous study integrated EEG and fNIRS to record brain activity during Stroop tasks (15), which initiated our study. The previous study focused on the relationship between brain hemodynamic and electrophysiological signals in the time dimension but lacked analysis of brain effective connectivity and functional connectivity. And the previous study only put 16 detectors which covered the left and right prefrontal cortex. Our system employed more channels, covered larger brain regions, and can obtain more information on brain activity. In future studies, the bimodal research approach can be applied to more cognitive functional paradigms, such as n-back tasks (48), go/no-go tasks (49), flanker tasks (50), and so on. Bimodal techniques can also be used for joint analysis of EEG and fNIRS brain networks, or to develop multimodal data processing algorithms.

In this study, we explored the human brain region that plays the role of an initiator, local information processing, and how information flow transfers between brain regions during processing the Chinese color-word match Stroop task. The frontal lobes especially the left frontal lobe play the leading role in dealing with conflict tasks. When processing the more complex incongruent stimulus, the human brain shows leftward lateralization, presented as the left frontal lobe showing stronger functional connectivity, and the information flow is from the left frontal lobe to the right frontal lobe. Therefore, our study promotes the understanding of brain activity during cognitive conflict processing.

## 5. Conclusion

In this study, we employed a Chinese color-word match Stroop task, significant Stroop effects emerged according to a decrease in accuracy, increase in reaction time, and decrease in accuracy/reaction time in the incongruent stimulus, which was more complex. The ERP component N200 and P300 also showed significant Stroop effects manifested as a delay in the time of occurrence and an increase in peak in the incongruent stimulus. We found that the frontal lobes especially the left frontal lobes played the leading role when the human brain processes cognitive tasks. Compared with processing the neutral stimulus, the human brain showed leftward lateralization when dealing with the more complex incongruent stimulus, the ability of local information processing in the left frontal region enhanced, and the information flow manifested as an increase in global flow from the left frontal lobe to the right frontal lobe. The above fNIRS results indicated that when the human brain deals with conflict events, information processing seems to be mainly carried out in the left hemisphere, especially the left frontal lobe. However, in EEG results there were no such finding, and there were no lateralization differences. In EEG data and fNIRS data there were lateralization differences in functional activity during Stroop tasks, EEG showed no lateralization while fNIRS showed left lateralization. Therefore, it is necessary to combine EEG signals and fNIRS signals to analyze brain activity during cognitive detect and processing more comprehensively. It is valuable to apply the dual modality method combining EEG and fNIRS to excavate more information through cognitive and physiological studies.

## Data availability statement

The original contributions presented in the study are included in the article/supplementary material, further inquiries can be directed to the corresponding author.

## Ethics statement

The studies involving humans were approved by the Clinical Research Ethics Committee of the First Affiliated Hospital, College of Medicine, Zhejiang University (IIT20210036C-R1). The studies were conducted in accordance with the local legislation and institutional requirements. The participants provided their written informed consent to participate in this study.

## Author contributions

ZC completed most of the data collection and processing, and independently wrote the initial manuscript. XJ made suggestions for data processing and made revisions to the entire manuscript. CG provided advice on manuscript revision. GL helped with EEG brain connectivity analysis. SL helped with part of the data collection. TL and YZ contributed to the conceptualization of the study and participated in the revision, editing, and final approval of the manuscript. All authors read and approved the final manuscript.

## Funding

This research was funded by the National Natural Science Foundation of China (grant no. 81971660), the Medical and Health Innovation Project (grant nos. 2021-I2M-1-042, 2021-I2M-1-058, and 2022-I2M-C&T-A-005), the Sichuan Science and Technology Program (grant no. 2021YFH0004), the Tianjin Outstanding Youth Fund Project (grant no. 20JCJQIC00230), and the Program of Chinese Institute for Brain Research in Beijing (grant no. 2020-NKX-XM-14).

## Conflict of interest

The authors declare that the research was conducted in the absence of any commercial or financial relationships that could be construed as a potential conflict of interest.

## Publisher’s note

All claims expressed in this article are solely those of the authors and do not necessarily represent those of their affiliated organizations, or those of the publisher, the editors and the reviewers. Any product that may be evaluated in this article, or claim that may be made by its manufacturer, is not guaranteed or endorsed by the publisher.

## Footnotes

^1^https://letswave.cn/

^2^http://noc.ac.uk/using-science/crosswavelet-wavelet-coherence
